# Increasing Awareness and Identification of Maturity-Onset Diabetes of the Young in a Pediatric Diabetes Program

**DOI:** 10.1097/pq9.0000000000000937

**Published:** 2026-10-28

**Authors:** Raymond J. Kreienkamp, Jessica L. Ruiz, Sarah Schlegel, Olivia Sterns, Jia Zhu, Tara Maxwell, Christine E. Cherella, Erinn T. Rhodes

**Affiliations:** From the * Division of Endocrinology, Boston Children’s Hospital, Boston, Mass.; † Department of Pediatrics, Harvard Medical School, Boston, Mass.; ‡ The Broad Institute of MIT and Harvard, Cambridge, Mass.; § Department of Pediatrics, Harvard Medical School, Boston, Mass.; ¶ Division of Endocrinology & Diabetes, Children’s Hospital of Philadelphia, Philadelphia, Pa.; ‖ University of Pennsylvania Perelman School of Medicine, Philadelphia, Pa.

## Abstract

**Introduction:**

Maturity-onset diabetes of the young (MODY) is frequently misdiagnosed, but an accurate diagnosis can inform tailored diabetes management. A quality improvement project was launched in 2022 at a pediatric tertiary care center to (1) increase MODY testing among individuals with islet autoantibody-negative (IAb−) new-onset diabetes mellitus (NODM) and (2) increase provider awareness of MODY.

**Methods:**

Interventions targeted the primary drivers, including provider recognition of MODY and barriers to testing. Each month, an electronic medical record report identified patients with IAb− NODM diagnosed within the prior 3 months. A standardized previsit email was sent to the treating provider that (1) flagged IAb− status, (2) outlined MODY testing criteria, and (3) provided instructions for ordering genetic testing when indicated. The primary outcome, MODY tests ordered among eligible patients, was analyzed longitudinally using a control chart. Provider awareness of MODY and perceptions of the intervention were assessed in May 2023 and March 2024.

**Results:**

The proportion of patients with IAb− NODM tested for MODY at a clinic visit within 365 days of diagnosis increased from 2.87% at baseline (January/February 2021–May/June 2022) to 9.94% after the quality improvement project launch in July/August 2022. Among completed tests through October 2025, 9 of 28 (32.1%) identified a pathogenic or likely pathogenic MODY variant. Providers’ familiarity with MODY testing criteria also improved, with those reporting being “extremely familiar” increasing from 0% (0/20) to 17% (3/18).

**Conclusions:**

The project, including the targeted previsit provider email, was associated with increased MODY testing and improved provider awareness in a pediatric diabetes clinic.

## INTRODUCTION

Maturity-onset diabetes of the young (MODY) is an important diabetes subtype caused by variants in a single gene. Causal variants disrupt appropriate insulin production, secretion, or action, leading to diabetes.^1^ Compared with individuals with type 1 diabetes (T1D) or type 2 diabetes (T2D), individuals with MODY often respond better to targeted therapies, are at risk for distinct comorbidities, and may not require treatment.^2^ Additionally, a MODY diagnosis has implications for family members, in whom establishing a MODY diagnosis may have similar benefits.^3^ Although identifying youth with MODY is critical for providing optimal care, greater than 80% of individuals with MODY may be misdiagnosed or incorrectly classified.^4^

A definitive diagnosis of MODY is made with genetic testing. Although there are guidelines outlining when genetic testing should be pursued,^2^ practical barriers lead to missed opportunities for testing eligible patients. Clinically, it can be challenging to differentiate individuals with MODY from the more common T1D and T2D.^5^ Providers are often less familiar with MODY than more common forms of diabetes and thus might not always consider this diagnosis.^6^ Islet autoantibody (IAb) testing results, which generally differentiate T1D from MODY,^7^ are not available at diagnosis and must be reviewed later, sometimes by a different clinician. Providers must also navigate substantial logistical and insurance barriers to complete genetic testing. Although MODY testing is cost-saving when used appropriately,^8^ insurance requirements have made genetic testing increasingly time-consuming and burdensome, discouraging some providers from pursuing testing. Indeed, prior studies demonstrate that genetic testing rates increase when providers receive greater institutional and administrative support.^9,10^

Given concern for unrecognized MODY, limiting access to tailored diabetes care, we conducted a quality improvement (QI) project beginning in July 2022 to improve recognition of and support for MODY in our pediatric diabetes clinic. The institutional framework used was adapted from Six Sigma, Lean, and the Model for Improvement, consisting of 4 processes (define, diagnose, test and implement, and sustain), and adhered to the SQUIRE 2.0 QI reporting standards.^11^ As part of this framework, the project was able to (1) describe the problem of “underdiagnosis” of MODY, (2) delineate the processes contributing to the problem, (3) design interventions to aid providers in identifying and testing for MODY, and (4) monitor for improvement through the quantification of the number of MODY tests sent by providers in our clinic.

The global aims of this QI initiative were to (1) increase MODY testing among individuals with IAb− new-onset diabetes mellitus (NODM) and (2) increase provider awareness of MODY. The initial SMART aim was to increase the percentage of MODY tests ordered by 30% from baseline by January 2024 for patients followed in the Boston Children’s Hospital Diabetes Program who have IAb− NODM that was diagnosed within the prior 12 months. The QI project activities continued beyond our initial planned timeline and are presented here through October 2025.

## METHODS

### Setting

Boston Children’s Hospital (BCH) is a pediatric tertiary care academic medical center in Boston, Massachusetts, with inpatient and outpatient diabetes care. The Diabetes Program at BCH, housed within the Division of Endocrinology, manages about 250–300 patients with NODM annually and has a population of approximately 2,500 outpatients with DM. The Division of Endocrinology has 36 physicians caring for patients with diabetes, 11 fellows, and 14 outpatient diabetes nurse educators. Each year, approximately 10,000 diabetes mellitus visits occur at BCH across 5 clinical sites for patients younger than 19 years of age.

### Planning the Interventions

In July 2022, a QI team consisting of pediatric endocrinology fellows (R.J.K., J.L.R., S.F.S.), endocrinologists (J.Z., T.M., C.E.C., E.T.R.), and a QI consultant (O.S.) at BCH identified a MODY prevalence in our program that was lower than might be expected based on the literature—1% in our program versus 2.5% to 6% in other settings^2^ — suggesting the possibility of unrecognized MODY. The team then identified barriers to MODY screening using a fishbone diagram (Fig. 1). Notable contributors included lack of familiarity with when to order MODY testing, logistical challenges associated with ordering genetic testing, and the time demand associated with ordering testing.

**Fig. 1.**
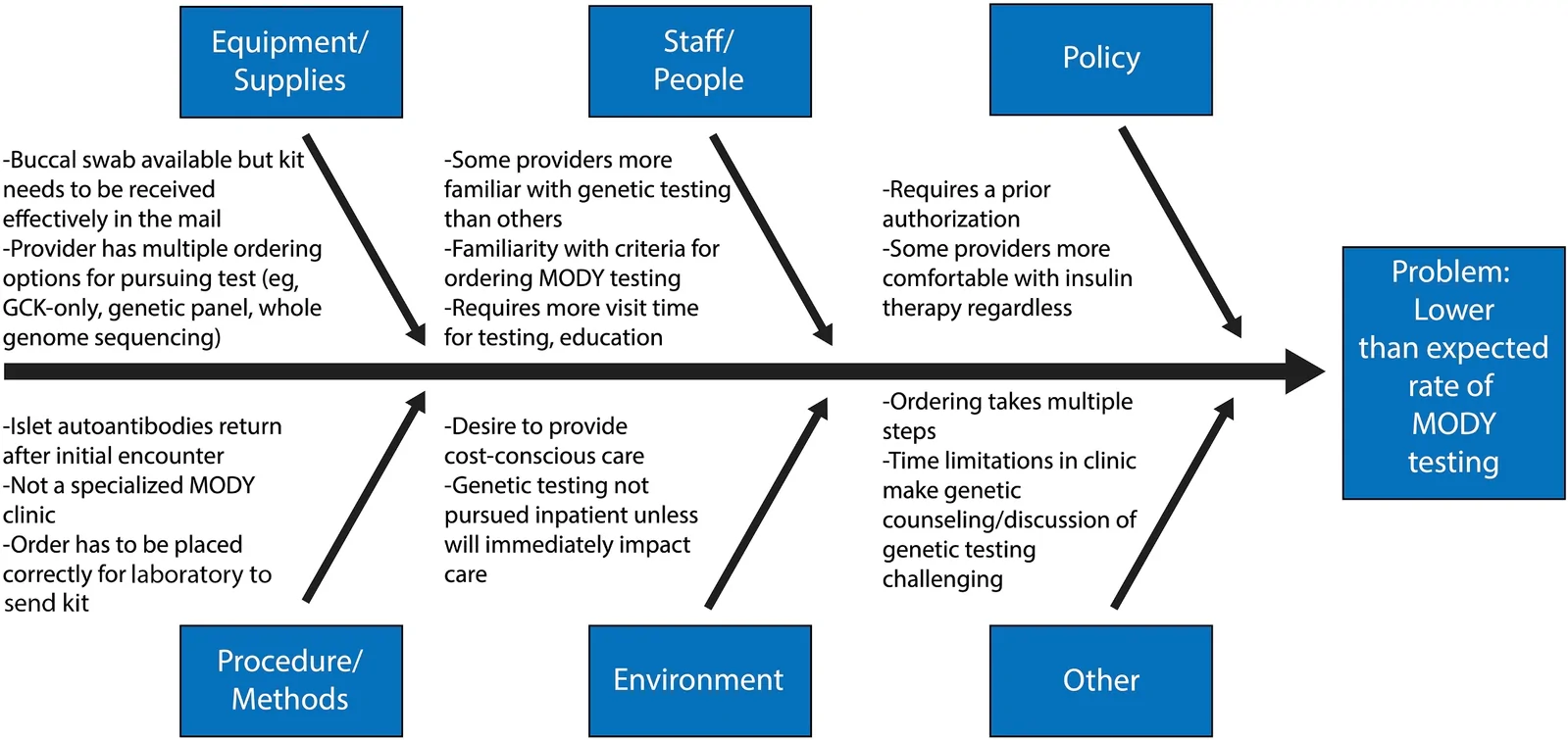
Fishbone diagram identifying potential contributors to a lower-than-expected rate of MODY screening.

A goal was established to (1) increase MODY testing among individuals with IAb− diabetes and (2) increase provider awareness of MODY. Based on this goal and identified obstacles, we identified interventions for improvement as outlined in our key driver diagram (Fig. 2). Specifically, we noted the importance of improving provider awareness of appropriate times to order testing, as well as providing instructions and support for how to order testing.

**Fig. 2.**
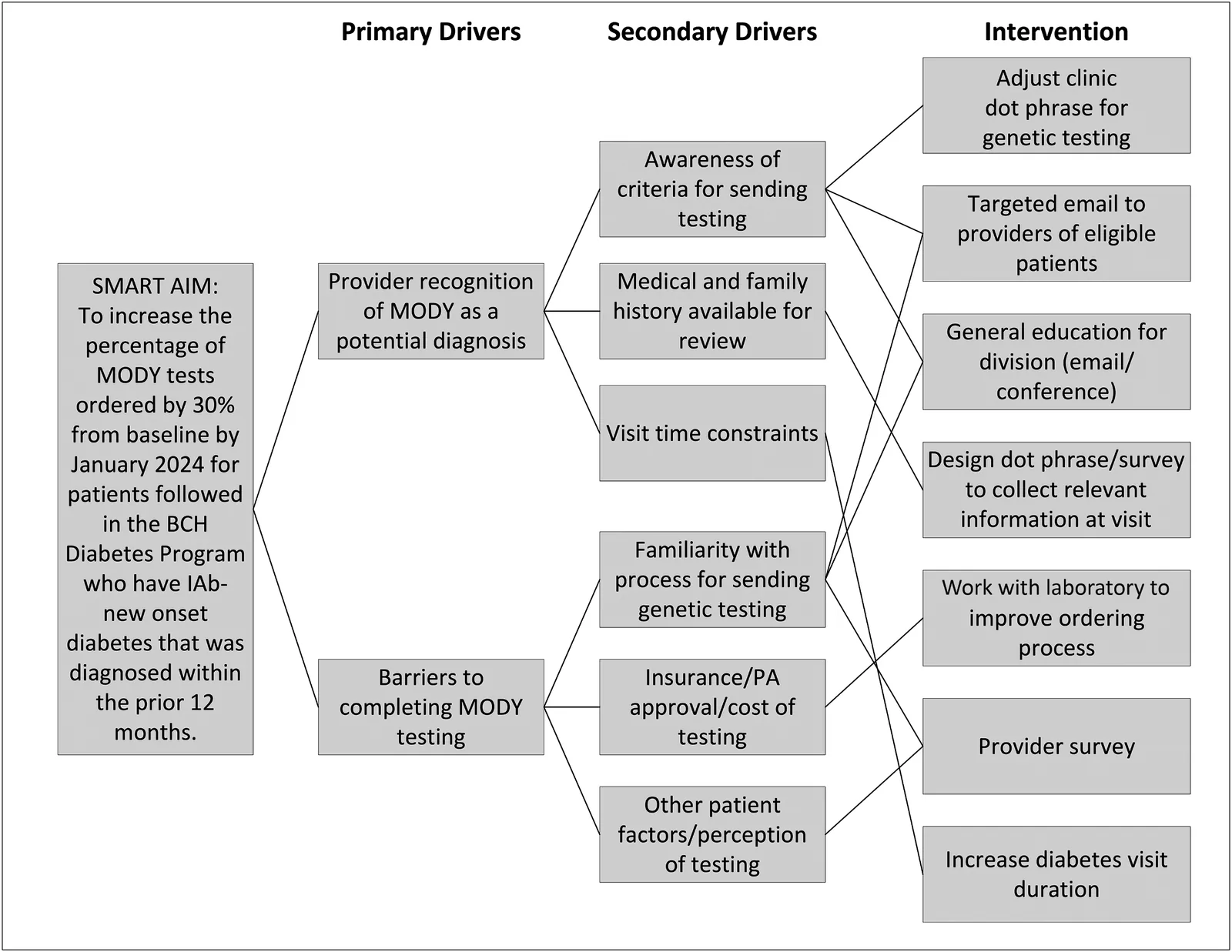
Key driver diagram.

### Interventions

Based on the planning activities initiated in July 2022, the QI project team developed interventions targeting the primary drivers. The team led multiple division-wide presentations to discuss this QI initiative (February 2023) and review genetic testing protocols (April 2023). Targeted discussion topics included criteria for sending MODY testing, availability of a dot phrase for use in the electronic medical record (EMR) (March 2023), proper ordering technique, and resources for prior authorization letters and for testing that was denied by insurance. All Plan-Do-Study-Act (PDSA) cycles are outlined in Table 1.

**Table 1.** Timeline of PDSA Cycle Interventions and Project Events

Project Event	Date	PDSA Cycle Intervention and Events
Baseline	January 2021–June 2022	Baseline period following introduction of routine IAb testing for all patients with NODM
Event 1	July 2022	QI project launch with baseline assessment and stakeholder engagement
PDSA 1	February 2023	Division-wide presentation to discuss MODY QI project
PDSA 2	March 2023	Dot phrase with reasons for MODY testing rolled out
	April 2023	Began sending monthly targeted provider emails
	April 2023	Division-wide presentation reviewing genetic testing
	April 2023	Prior authorization templates generated for providers
PDSA 3	May 2023	REDCap survey 1 sent to the division and email tools revised
PDSA 4	March 2024	REDCap survey 2 sent to the division and email tools revised
Event 2	June 2024	EMR transition
PDSA 5	June 2024	MODY ordering process changed. Temporary pause in targeted emails due to challenges collecting IAb testing metrics
PDSA 6	September 2024	Restarted sending monthly targeted provider emails
PDSA 7	April 2025	Updated targeted provider email with new ordering processes
Event 3	July 2025	BCH Diabetes Genetics Clinic established
PDSA 8	August 2025	Additional changes made to genetic testing ordering process

In April 2023, the project team initiated scheduled, templated emails to diabetes care providers to consider MODY testing for eligible patients, which was subsequently refined by PDSA cycles using provider feedback and survey data. To identify appropriately eligible patients, a monthly EMR report was generated for patients diagnosed with IAb− NODM in the prior 3 months with an appointment scheduled in the reporting month. We included all individuals with IAb− NODM, including those with features suggestive of T2D, because MODY can present with a T2D-like phenotype^12^ and is often difficult to distinguish clinically from T2D.^13^

For each patient identified, a standardized previsit email was sent by one of the 3 project leads to the scheduled clinical provider 2 business days before the patient’s scheduled clinic visit. The email template noted the IAb− status, outlined MODY testing criteria, and provided instructions for ordering MODY testing when indicated. (**See Supplemental Digital Content 1**, which demonstrates the standardized previsit email template, https://links.lww.com/PQ9/A829.) MODY testing criteria included guidelines provided by the International Society of Pediatric and Adolescent Diabetes,^2^ including diabetes diagnosed in the first year of life, an unusual clinical phenotype, a family history of diabetes without typical features, a family history of MODY, extra-pancreatic features, and atypical findings among those with a T2D phenotype (eg, lack of severe obesity or unusual fat distribution). A prior authorization template and optional “dot phrase” for use as standardized documentation in the EMR clinic note were also provided to assist with insurance approval for these tests. The email also included contact information for members of the QI team for additional troubleshooting, as well as additional considerations for patient management in these cases, including research studies such as the Rare and Atypical Diabetes Network (RADIANT).^14^

### Study of the Interventions

MODY tests ordered among eligible patients were tracked and analyzed on a statistical process control P-chart (Fig. 3). Provider awareness of MODY and challenges to sending MODY testing were assessed using a REDCap survey in May 2023. A follow-up survey in March 2024 also assessed perceptions of the interventions.

**Fig. 3.**
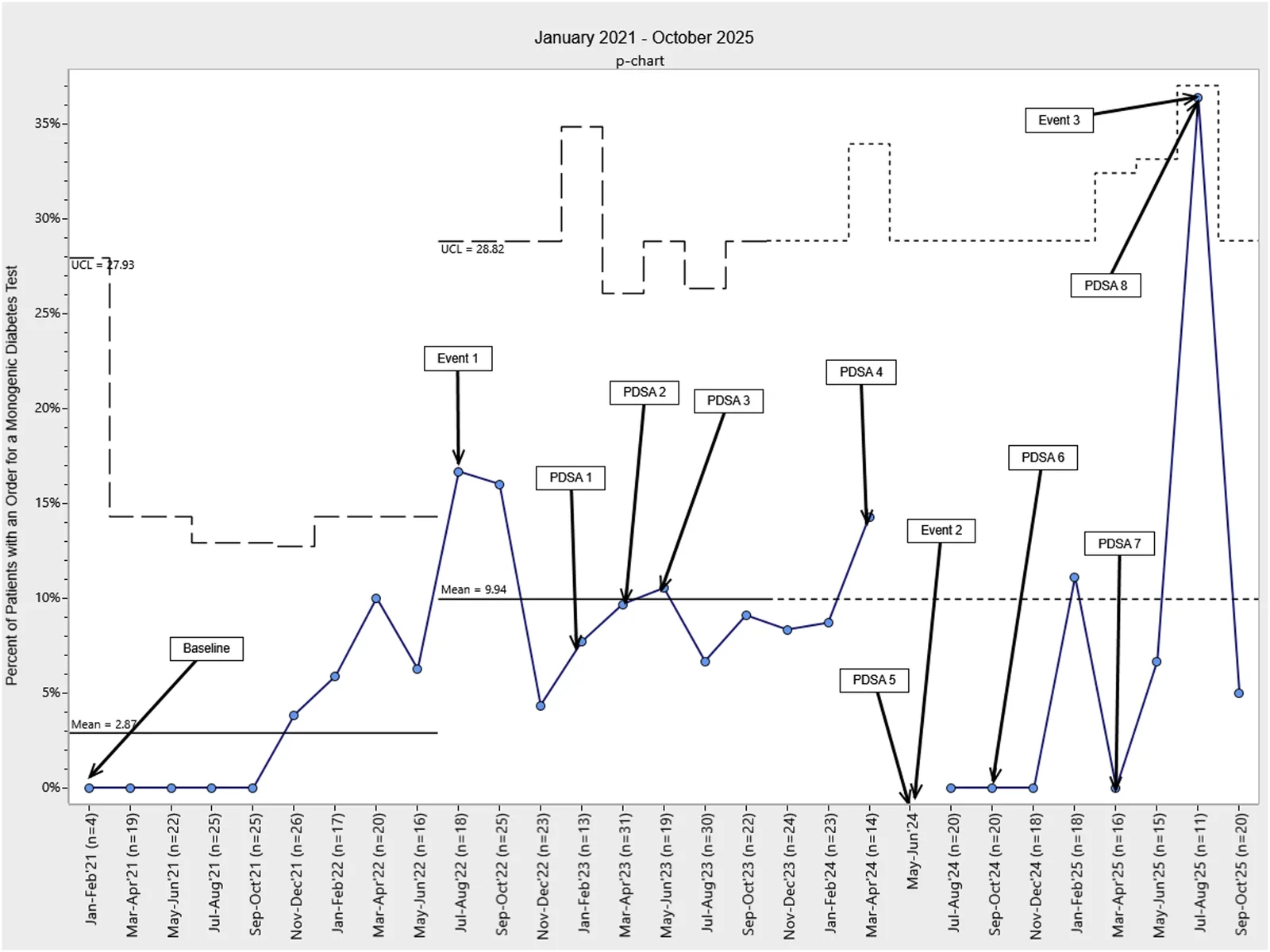
Statistical process control P-Chart displaying the percentage of monogenic diabetes tests ordered among IAb− patients with new-onset diabetes diagnosed within 365 days of the visit date. A higher percentage represents improved performance. The baseline period was January/February 2021–May/June 2022. Details of the PDSA cycles and events are presented in Table 1. The center line (mean) is fixed beginning in September/October 2023, as represented by the dashed line. UCL, upper control limit.

### Measures and Analysis

The primary outcome was the percentage of MODY tests ordered—defined as the number of patients with MODY testing ordered among IAb− patients diagnosed with diabetes within 365 days of the visit date and followed in the BCH Diabetes Program. Process measures included (1) the percentage of MODY tests resulted among eligible patients for whom the test was ordered and (2) provider knowledge of criteria for MODY testing and likelihood of sending MODY testing. Perceived burden of ordering MODY testing was evaluated by provider survey as a balancing measure.

Analysis of the interventions was conducted throughout the project. Baseline data were reviewed before the project launch. A provisional baseline was calculated using observations from January/February 2021 to May/June 2022, before the launch of any project activities. Subsequently, data were collected and analyzed iteratively. The primary outcome measure was evaluated with statistical process control on a p-chart, with established rules to identify special cause variation.^15^ Provider knowledge and practices were evaluated with a survey sent to BCH endocrine clinical faculty and endocrine fellows in May 2023 and again in March 2024 to assess provider awareness of MODY and the impact of the interventions (process measure—provider knowledge) as well as potential for provider burden (a balancing measure). The surveys were developed and circulated to the providers using REDCap.^16^

### Ethical Considerations

This project was deemed consistent with the QI project guidelines of the BCH Department of Pediatrics following review by its Performance Excellence Group and was exempt from institutional review board review.

## RESULTS

The proportion of MODY tests ordered among eligible patients was tracked and analyzed on a statistical process control P-chart spanning from January 2021 to October 2025 (Fig. 3). Interventions and events are annotated on the control chart and further explained in Table 1. Among IAb− patients with NODM diagnosed within 365 days of a visit, the average percentage of MODY tests ordered was 2.87% during the baseline period (January/February 2021–May/June 2022). Following the launch of the QI project in July/August 2022, there were 8 consecutive observations above the baseline mean, consistent with special cause variation and reflecting a sustained shift. The new mean of 9.94% was recalculated using these data from July/August 2022 to September/October 2023 and then held for ongoing monitoring. This represented a 3.5-fold (246%) increase in MODY testing. MODY testing was disrupted following the EMR transition (May/June 2024), with resumption of MODY test ordering in January/February 2025 (Fig. 3).

Between July 2022 and October 2025, 28 out of 32 (88%) of MODY tests ordered were ultimately completed. The remaining 12% that were canceled or not completed were due to insurance-related reasons. Of the 28 MODY tests with results since the project launched, 9 tests (32.1%) had likely pathogenic or pathogenic variants, all of which were associated with changes in clinical management (Table 2). There were 6 additional individuals with variants of uncertain significance, including one associated with changes in clinical management (Table 2). Iterative PDSA cycles addressed provider feedback in response to the targeted reminders and to surveys and focused on provider suggestions to improve the ease of ordering testing and decrease the likelihood of denials for clinically indicated genetic testing.

**Table 2.** Changes in Clinical Management Based on Genetic Testing Results

Diagnosis After Genetic Testing (n)	Effect on Clinical Management
*GCK*-MODY (6)	• All individuals stopped pharmacotherapy • Four of six individuals no longer required routine endocrine follow-up • One individual continued care for obesity • One individual transitioned care to another institution; therefore, it is unknown if routine endocrine follow-up is occurring
*HNF1A*-MODY (2)	• Two individuals transitioned from basal-bolus insulin to a sulfonylurea plus daily long-acting insulin
*HNF1B*-MODY (1)	• One individual had no change in pharmacotherapy but underwent a renal ultrasound that resulted in actionable findings
Variant of uncertain significance in a MODY gene (6)	• One individual transitioned from insulin to a glucagon-like peptide-1 receptor agonist • Two individuals transitioned care to another institution; therefore, it is unknown if management changed • Two individuals had no change in pharmacotherapy or management • One individual developed positivity in 2 IAb after being on insulin

### Surveys

In addition, provider awareness of MODY and response to the email intervention were tracked with 2 division-wide surveys of physicians (May 2023, responses n = 20 [32.8%]; March 2024, responses n = 18 [29.5%]). These surveys specifically assessed familiarity with criteria for MODY testing and the likelihood of sending MODY testing. In the initial survey, no providers noted that they were “extremely” familiar with criteria for sending MODY testing. However, in the follow-up survey, the number of providers who stated that they were “extremely” familiar with these criteria increased to 17% (3/18), and all survey respondents who had received an email (n = 6 providers) acknowledged that the email increased their likelihood of sending MODY testing. Indeed, 50% (3/6) of these individuals stated that the email increased their likelihood of sending MODY testing “quite a bit” or “extremely.” Providers reported that the email helped clarify when to consider MODY testing, how to order MODY testing, how to obtain preauthorization, and how to proceed if denied by insurance. One provider noted that the QI project “motivated me to consider monogenic diabetes testing in more patients.” Survey respondents reported that other project interventions were also helpful. For example, 8/10 (80%) of individuals who reported exchanging emails with the QI project team—whether initiated by the targeted previsit email or independently by the provider—noted that the communication increased their familiarity with MODY testing. Additionally, among survey respondents who reported attending the division presentation, 87% (13/15) reported that it increased their familiarity with MODY testing.

Although the survey highlighted some successes of the project, it also highlighted further opportunities for improvement and the potential burden of MODY testing (balancing measure). One provider noted, “[t]he challenges involved in obtaining pre-authorization are discouraging.” Consistent with this, in the March 2024 survey, 44% (8/18) of individuals responded that they were “unlikely” or “extremely unlikely” to pursue additional testing after initial insurance denial, whereas only 28% (5/18) of individuals indicated that they would continue to pursue testing despite a denial. In response, these findings informed additional PDSA cycles implemented after the EMR transition to support sustainment of the project, including adding insurance support resources to the provider email and support for establishing a Diabetes Genetics Clinic to assist with authorization and testing when insurance barriers arise.

Additionally, some providers noted that the email could be improved if targeted to individuals with normal body mass index. One provider wrote, “Reminders are sent on all antibody negative patients, many of whom clearly have type 2 diabetes, perhaps emails can be restricted to patients without abnormally high BMI.” However, we opted to continue sending to all individuals with IAb− NODM given that of the 9 individuals found to have confirmed MODY, 2 were overweight or obese. Each of these individuals had a 3-generation family history of diabetes, and neither had acanthosis nigricans. In one of these cases, the provider noted that the QI project “played a role in…thinking about [genetic testing] and sending it.”

## DISCUSSION

This QI project demonstrated that efforts to support MODY screening, including a targeted monthly email to providers of individuals with IAb− NODM and division-wide presentations about MODY, were associated with higher MODY testing rates and improved provider awareness at a pediatric tertiary care academic medical center. Among patients with IAb− NODM diagnosed within 365 days of a visit, the average percentage of MODY tests ordered increased from 2.87% at baseline (January/February 2021–May/June 2022) to 9.94% following the launch of the QI project in July/August 2022, a 3.5-fold (246%) increase, well above the stated goal of a 30% increase. Testing during the project resulted in improved clinical care: 32.1% of all MODY tests completed returned with a pathogenic or likely pathogenic MODY variant, all of which changed clinical management. Following these interventions, providers reported an increased familiarity with MODY and an increased likelihood of sending MODY testing in the future.

Importantly, a change in the EMR occurred during the project timeline. This was associated with a temporary decrease in MODY testing, likely due to changes in the test ordering process. In response, we updated the email template, and the division received additional presentations on genetic testing workflows. In July/August 2025, MODY testing had increased to the highest level observed over the course of the entire project and, since January/February 2025, appeared more consistent with testing before the EMR transition, suggesting that the intervention may be sustainable despite this disruption.

To our knowledge, this is the first clinic-based QI initiative to include an EMR-based targeted email to consider MODY testing in youth-onset IAb− NODM. Previous studies have demonstrated that clinical tools, such as the MODY probability calculator,^17^ or the genetic diabetes nurse project, which increase provider awareness of MODY,^18^ can improve identification of MODY. This intervention aligns well with interventions in other fields to increase genetic testing. A systematic review of genetic testing in routine oncology care demonstrated that interventions with multiple components, such as provider education and a documentation or system change, increased genetic testing completion by 15% on average.^19^ A neonatology QI project increased rapid genome sequencing among eligible neonatal intensive care unit infants from 37% preintervention to 54% postintervention with census review, provider education, and simplified ordering.^20^

This intervention could be readily implemented in other practices, as it requires only a standardized review of IAb results among patients with NODM, a templated email prompting the accepting provider to consider MODY testing, and a description of the process to order MODY testing. Although this intervention required dedicated staff time for monthly data review and individualized emails, future iterations could leverage EMR-based tools such as best practice alerts to automate patient identification and provider notification, reducing the ongoing effort required. This intervention has the potential for significant clinical impact, as exemplified by the observed changes in clinical care.

We note several limitations. First, this initiative was conducted at a single institution and a large academic medical center. Familiarity with MODY and genetic testing at this institution may differ from that at other clinics. Additionally, the BCH laboratory regularly sends genetic testing for a variety of conditions, with institutional support for the PA process. In some clinics, even with our targeted intervention, a lack of genetic testing infrastructure may limit the effectiveness of the intervention. There was also a transition to a new EMR, which temporarily disrupted testing workflows. Thus, our results may underestimate the impact of this intervention in settings without such disruptions. Finally, our baseline was provisional. However, a sustained shift was demonstrated starting with the launch of the project.

Additionally, this intervention focused on individuals with IAb− NODM. Our center began routine IAb testing in late 2020, aligned with our selected baseline period. In some centers, IAb testing is not routinely obtained as part of NODM care, making a similar intervention to ours unfeasible. However, given the importance of IAb testing in distinguishing diabetes type,^21^ it is likely that more centers will have access to these data as routine IAb testing increases.

The provider surveys were helpful in tracking provider awareness of MODY, as well as the impact of our interventions. However, participation in these surveys was incomplete, with 20 (32.8%) and 18 (29.5%) of a total of 61 possible respondents, respectively, responding, with not all of the same individuals responding to each survey. Additionally, only 6 individuals who received the targeted email responded to the survey. Therefore, it is possible that the survey results were biased and incompletely characterized the effect of this intervention, either in a positive or negative manner.

Despite our successful increase in screening, the overall screening remains low. Providers may determine that clinical findings (eg, convincing evidence for a diagnosis of T2D) or history may not support the need for screening among some potentially eligible patients. Our findings also suggest that the availability of genetic testing and support for insurance coverage and authorization may also be barriers. One focus for ongoing PDSA cycles will be to support coverage for genetic testing after insurance denial. As noted, only 88% of test orders resulted; the remainder were canceled or incomplete due to insurance. Future PDSA cycles, including the expanded use of the Diabetes Genetic Clinic, will work to increase genetic testing covered by insurance. Further financial support and testing options might ultimately lead to an increased rate of successful testing.

## CONCLUDING SUMMARY

This project provides a framework to increase MODY testing among individuals with IAb− NODM by improving provider familiarity with testing criteria and implementing a standardized intervention to facilitate test ordering. The project resulted in improved clinical care and increased provider awareness of MODY. Additional PDSA cycles will focus on increasing resources to address insurance denial of MODY testing in clinically indicated settings.

## ACKNOWLEDGMENTS

The authors thank Katharine Garvey, MD, MPH, and Gretchen Waldman, MSN, RN, CDCES, for their support of this project. The authors also thank all providers in the BCH Division of Endocrinology for their engagement in this project.

## Supplementary Material
