# Supplementary material for: Increasing Awareness and Identification of Maturity-Onset Diabetes of the Young in a Pediatric Diabetes Program
Source: Pediatr Qual Saf. 2026 Oct 28;11(6):e937. doi: 10.1097/pq9.0000000000000937 (PMC13716420; doi:10.1097/pq9.0000000000000937)
Supplement: Supplementary file 1 [file pqs-11-e937-s001.pdf]

Standardized Pre-Visit Email Template

Hello,

We are emailing you because you are the primary diabetes provider(s) for XXXXXX (MRN XXXXXX), who was recently diagnosed with diabetes, with negative islet autoantibody testing. You are scheduled to see this patient on XXXXXX at XXXXXX AM/PM.

While 5-15% of patients with type 1 diabetes are autoantibody negative, and many patients with type 2 diabetes are autoantibody negative, patients with maturity-onset diabetes of the young (MODY) are also generally autoantibody negative. Given that your patient is autoantibody-negative, we would encourage you to **consider sending MODY testing if you assess that it is clinically indicated**, as diagnosis of a patient with MODY can improve clinical care and change treatment options.

Below, we have outlined reasons to send MODY testing and how to send MODY testing via either **blood or buccal swab** specimen.

Reasons to send MODY testing include:

1. Patient diagnosed with diabetes in the first year of life
2. Patient with unusual clinical phenotype and/or abnormal insulin regimen needs
3. Family history of diabetes without typical features:
    1. Family members with autoantibody negative diabetes
    2. Family members with unusual trajectory
4. Family history of MODY
5. Extra-pancreatic features
    1. Congenital heart or gastrointestinal defects
    2. Brain malformations
    3. Severe diarrhea
    4. Other autoimmune conditions in a young child
6. In those matching type 2 diabetes picture:
    1. Lack of consistent severe obesity
    2. Lack of or particularly severe acanthosis nigricans
    3. Unusual fat distribution, particularly with lack of subcutaneous fat
    4. Dyslipidemia

To send genetic testing:

1. Order "Mody Panel"
2. Fill out order details as below:
    1. Specimen Type:
        i. You may order as a usual blood test, if patient is having blood ordered. Specimen Type: “Blood”
        i. If blood draw not otherwise planned, we recommend remote-buccal option, where patient will be shipped a kit. Please inform them to look to receive kit in mail. Select - Specimen Type: “Swab”
    1. Remote Collection - select "Yes" if you had selected "Swab"
    2. Clinic indication - add a short statement about why sending testing (e.g. "pancreatic autoantibody-negative diabetes in individual without acanthosis nigricans, not matching phenotype of type 2 diabetes").
    3. Family History - add relevant details
    4. Billing - "insurance"

3. If you would like to save a letter of medical necessity to the chart, you can steal a templated letter dotphrase from XXXXXX (MODYLOMN). Please add the relevant details for your specific patient. You may also add the following dotphrase XXXXXX to your clinic note for documentation within your clinic note.
4. Testing will then proceed. If you run into any problems/there are any issues, please contact us.

If you have any challenges ordering or would like someone else to order, you can offer them a one-time visit in the Diabetes Genetics Clinic, where they can be seen for one virtual visit to help with genetic test ordering.

If you run into any problems or concerns with ordering, please contact us.

Once tests return, if uncertain of interpretation, please contact us, at which point we can connect you with additional resources. If testing is negative, you might consideration enrollment in RADIANT study (https://www.atypicaldiabetesnetwork.org/).
